# Diane-35 and Metformin Induce Autophagy and Apoptosis in Polycystic Ovary Syndrome Women with Early-Stage Endometrial Carcinoma

**DOI:** 10.3390/genes13010131

**Published:** 2022-01-12

**Authors:** Yanjun Liu, Yang Wang, Dan Yao, Xing Chen, Feifei Zhang, Yi Feng, Xin Li

**Affiliations:** 1 Department of Obstetrics and Gynecology, Shanghai Medical School, Fudan University, Shanghai 200011, China; yanjunliu18@fudan.edu.cn; 2 Shanghai Key Laboratory of Female Reproductive Endocrine Related Diseases, Fudan University, Shanghai 200011, China; 3 Department of Gynecology, Obstetrics and Gynecology Hospital of Fudan University, Shanghai 200011, China; wangyangdeyouxiang@163.com (Y.W.); xing_chen@shmu.edu.cn (X.C.); feifeizhang@fudan.edu.cn (F.Z.); 4 Department of Endocrinology, Xiangshan Hospital of TCM Medical and Health Group, Ningbo 315700, China; yaodan1218@126.com; 5 Department of Integrative Medicine and Neurobiology, School of Basic Medical Sciences, Institutes of Brain Science, Brain Science Collaborative Innovation Center, State Key Laboratory of Medical Neurobiology, Fudan Institutes of Integrative, Shanghai 200011, China

**Keywords:** Diane-35, metformin, autophagy, polycystic ovary syndrome, early-stage endometrial carcinoma

## Abstract

Objective: Women with polycystic ovary syndrome (PCOS) are at increased risk ofendometrial carcinoma (EC). Previous studies indicated that the combined therapy of Diane-35 and metformin significantly suppresses disease progression in PCOS patients with early EC; however, the mechanisms remain unclear. Methods: An established murine model of PCOS with early EC, clinical specimens, and human EC cells was used in this study. The levels of protein and mRNA were measured with Western blotting and RT-PCR, respectively. Cell proliferation was determined with MTT, colony formation, and flow cytometry. Proteins were analyzed with immunofluorescence and immunohistochemistry. Results: Diane-35 and metformin significantly inhibited proliferative activity and promoted apoptosis in EC cells. Additionally, cell autophagy was induced by the combined therapy. Quantitive PCR revealed that Diane-35 and metformin decreased androgen receptor (AR) expression but elevated GLUT4 expression. AR was found to repress GLUT4 expression by binding to the promoter of GLUT4. Moreover, the combined treatment mediated the onset of cellular autophagy by regulating the mTORC pathway via the suppression of IGF-1 and inhibited the development of EC by the activation of the PI3K/mTORC pathway. Conclusion: The results and previous clinical evidence support the use of Diane-35 and metformin combination therapy for patients with PCOS and early EC.

## 1. Introduction

Polycystic ovary syndrome (PCOS) is associated with a greater risk of endometrial carcinoma (EC) and is accompanied by elevated levels of androgens, ovulatory arrest, and characteristics of metabolic syndrome, such as insulin resistance and obesity [1,2,3]. The pathophysiology of PCOS is characterized by the abnormal secretion of gonadotropin in response to reduced hypothalamic activity as a consequence of functional changes in the ovaries and insulin dysregulation [4]. The exact etiology of PCOS is not clear but could be a combination of genetic disposition and environmental factors [5,6]. However, the more serious consequence of PCOS is its association with EC [7]. Women with PCOS are three times more at risk of EC than women without [8] and more likely to be diagnosed with EC at a later stage [9]. This is a concern, because the survival rate of EC depends on the stage and classification of the disease [10]. In advanced disease, total hysterectomy is the predominant treatment. However, preventative measures include the control of obesity and diabetes; therefore, alleviating PCOS may also reduce the risk of EC [11,12].

The combination of Diane-35 and metformin was reported to significantly improve insulin resistance and suppress the progression of EC [13]. However, the mechanisms remain unclear. Diane-35 is a medicine containing progestogen (2 mg, cyproterone acetate) and estrogen (35 μg, ethinyl estradiol), which works by blocking the action of androgens (such as testosterone) and activating progesterone receptors in patients with hyperandrogenemia and PCOS. Metformin is a biguanide antihyperglycemic agent used in the treatment of type 2 diabetes and PCOS [14]. After treatment with metformin, the sensitivity to insulin increases, and glucose production by the liver is reduced [15]. However, its precise mode of action is unclear. It is believed to ameliorate glycemia through the adenosine monophosphate (AMP)-activated protein kinase (AMPK) pathway [16], but it may also act independently of AMPK through the inhibition of mitochondrial function [17]. A recent study proposed that the combination of metformin and Diane-35 could also improve ovulation in a rat model of PCOS through the glycolysis pathway [18].

Hyperandrogenemia and insulin resistance are both common characteristics of PCOS [19]. Glucose transporter 4 (GLUT4) is an insulin-regulated glucose transporter that is downregulated in PCOS [20]. Metformin can reverse the downregulation of endometrial GLUT4 expression in PCOS patients by changing the expression of the androgen receptor (AR) in response to reduced levels of androgen [21], subsequently, which brought about subsequent changes in the insulin receptor/PI3K/Akt/mTOR signaling pathway. Hyperandrogenism in PCOS is thought to be related to increased levels of androgen production by insulin and an upregulation of insulin-like growth factor (IGF-1) receptors. The upregulation of IGF-1 in the endometrium is also associated with the increased occurrence of EC in PCOS [22]. It has been proposed that elevated levels of IGF-1 may promote EC in patients with type 2 diabetes through increased levels of PI3k/CCND1-dependent cell growth [23]. 

Metformin can promote autophagy and apoptosis in cancer cells [24] and inhibit the proliferation of myeloma cells by inducing autophagy and G0/G1 phase cell cycle arrest through targeting the AMPK/mammalian target of the rapamycin complex (mTORC) pathway [25]. The inhibition of autophagy by mTORC1 in cancer cells is activated by the cancerous inhibitor of PP2A (CIP2A), which is overexpressed in EC [26,27]. In this study, we also investigated the influence of autophagy on cancer progression through the CIP2A/mTORC pathway. Therefore, based on this evidence and that from previous clinical reports [13,28], we further investigated the mechanism of Diane-35 and metformin combination therapy in PCOS women with EC.

## 2. Materials and Methods

### 2.1. Specimens

Tissue samples were obtained from PCOS women with early EC. Patients were administrated Diane-35 (Bayer Schering Pharma AG, Berlin, Germany) and metformin (Sino-American Shanghai Squibb Pharmaceuticals Ltd., Shanghai, China) for 2–6 months (one coated tablet dose of Diane-35 and 1000 mg of metformin daily). The therapeutic options were detailed in a previous submission [13]. Tissue specimens were obtained from patients with EC who had undergone a hysterectomy (*n* = 14). Endometrial tissues were stored in formalin-fixed solution or liquid nitrogen.

### 2.2. Cell Line and Culture

The human EC cell line (Ishikawa, CL-0283) was obtained from Procell Life Science &Technology (Wuhan, China). The cells were cultured in 90% phenol red-free DMEM (21063029, Gibco, Waltham, MA, USA), 10% charcoal dextran-treated FBS, and 1% P/S (PB180120) at 37 °C with 5% CO_2_. Cells were treated with Diane-35 (Bayer Schering Pharma AG) and metformin (Sigma-Aldrich, St. Louis, MO, USA, CAS:1115-70-4). The Diane-35 tablets were milled into a powder using a crucible, then dissolved in DMSO, centrifuged in an ultracentrifuge (5000× *g*, 15 min), and the supernatant was taken and prepared into 1/2/5/10-mM solutions for cell treatment. Metformin was prepared to the same concentrations using DMSO.

Ovarian granulosa cells were isolated from the ovaries of model mice. A PCOS and EC mouse model was constructed by the intraperitoneal injection of dehydroepiandrosterone (DHEA) and tumor cell transplantation (for further details, refer to the in vivo experiments section). Preantral follicles and small antral follicles were then punctured with a sterile puncture needle under a microscope (avoiding puncturing large sinus follicles). Ovarian granulosa cells were maintained in 90% DMEM (21063029, Gibco), 10% treated FBS, and 1% P/S (PB180120) at 37 °C with 5% CO_2_.

### 2.3. Quantitative Real-Time Polymerase Chain Reaction (qRT-PCR)

Total RNA was isolated using Trizol reagent (Invitrogen, New York, NY, USA) and transformed into cDNA using a cDNA synthesis kit (Applied Biosystems, Foster City, CA, USA). Quantitative real-time (qRT)-PCR analysis was performed using SYBR Green with a real-time PCR apparatus (Applied Biosystems). Primers were synthesized and purchased by Integrated DNA Technologies (Coralville, IA, USA). Primer sequences are listed in Table 1.

### 2.4. Western Blot Analysis

Western blot was performed as follows. Proteins were isolated from samples and cultured cells and extracted in RIPA solution (Biotechnology, Shanghai, China) supplemented with protease inhibitors (Roche, Basel, Switzerland). Nuclear and cytoplasmic protein fractions were obtained using a Nuclear and Cytoplasmic Protein Extraction Kit (Thermo Fisher Scientific, Waltham, MA, USA) according to the manufacturer’s instructions. Protein concentrations were determined by using a BCA assay (Pierce, Waltham, MA, USA). Equal amounts of proteins were separated by SDS-PAGE and then transferred to polyvinylidene fluoride (PVDF) membranes (Millipore, Burlington, MA, USA). Blots were subsequently incubated with primary antibodies against the following proteins: cyclin D (Invitrogen, PA5-29466), CDK2 (Abcam, Cambridge, MA, USA, ab32147), AR (Invitrogen, PA1-110), GLUT4 (Invitrogen, PA5-23052), IGF-1 (Invitrogen, PA5-19382), IGF-1R (Invitrogen, MA5-13817), LC3 (Invitrogen, PA1-16931), cleaved caspase-3 (Abcam, ab13847), CIP2A (Invitrogen, MA1-46001), PP2A (Abcam, ab32141), c-Myc (Abcam, ab32072), AKT (Abcam, ab38449), PI3K (Abcam, ab140307)), mTORC1 (Invitrogen, PA5-34663), and S6K (Invitrogen, MA5-15202), then incubated with secondary antibodies bound to horseradish peroxidase. Immunoreactivity was visualized using a chemiluminescent ECL Western Blot system (Millipore). ImageJ was used for the Western blots quantitative analysis.

### 2.5. CCK-8 Assay

Cell viability was measured at 24, 48, 72, and 96 hours using Cell Counting Kit-8 (CCK8, Sigma-Aldrich; No. 96992) according to the manufacturer’s protocols. Briefly, 20 μl of MTS reagent was added to the cells in 100 μl of medium in a 96-well plate and then incubated at 37 °C. The absorbance was read at 490 nm by a UV spectrophotometer (UV-8000T, Shanghai Metash Instruments, Shanghai, China).

### 2.6. Colony Formation Assay

After cell counting, Ishikawa cells were seeded at a low density (1000 cells/plate) and incubated in a humidified incubator at 37 °C for 10 days until visible colonies appeared. Cell colonies were stained with 0.5% Giemsa, and the colonies were counted. The colony formation rate = (number of colonies/number of inoculated cells) × 100%.

### 2.7. Flow Cytometry

Flow cytometry was performed 24 hours after transfection or treatment to analyze cell apoptosis. Harvested cells were washed with cold PBS and stained with 7-AAD and Annexin-V-FITC and analyzed for apoptosis using an Annexin V-FITC/7-AAD Kit (Beckman Coulter, Brea, CA, USA) according to the manufacturer’s protocols. Immediately after staining, cells were analyzed with a flow cytometer (Cell Lab Quanta SC; Beckman Coulter).

### 2.8. Immunofluorescence Staining

Ishikawa cells were seeded on a coverslip of a 24-well cell culture plate, fixed with 2% paraformaldehyde for 20 min, permeabilized with PBS containing 0.1% Triton X100 (PBS-T) for 5 minutes, and then blocked with 3% BSA in PBS-T. Immunostaining was performed using the primary antibodies. After incubation with the appropriate secondary antibodies, cells were washed with PBS, and the nuclei were stained with DAPI.

### 2.9. Dual-Luciferase Reporter Assay

The wild-type GLUT4 5′UTR target sequence containing the AR-binding site was cloned in the pmiRGLO luciferase vector (Promega, Madison, WI, USA) downstream of the luciferase gene. The mutant GLUT4 5′UTR sequence was cloned in the same vector. The primers for cloning were synthesized from Invitrogen. Control/wt GLUT4/mutant GLUT4 5′-UTR constructs (0.2 μg) were transfected into A549 cells cultured in 24-well plates. After 48 h, the cells were harvested and assayed using a dual-luciferase reporter assay system according to the manufacturer’s protocols (Promega) for the measurement of firefly and Renilla luciferase activities. Firefly luciferase and Renilla luciferase activities were standardized.

### 2.10. Chromatin Immunoprecipitation (ChIP)

ChIP assays were performed with the ChIP kit (Cell Signaling Technology, Danvers, MA, USA) following the manufacturer’s guidelines. Briefly, Ishikawa cells were incubated with dimethyl 3,3′-dithiobispropionamide-HCl (5 mM) at room temperature for 10 min, then washed with Tris-HCl (100 mM, pH 8.0). Next, they were incubated with formaldehyde (1%) for 10 min. The precipitated DNA samples were quantified by PCR. Data were presented as a percentage of input DNA.

### 2.11. Immunohistochemistry Assay

The samples were fixed in 10% phosphate-buffered formaldehyde for 24 h at room temperature, then embedded in paraffin and sectioned (4–6 μm). Expression of AR, GLUT4, IGF-1, and Ki67 at the tumor foci was detected in the uterine region by immunohistochemical staining. AR polyclonal rabbit antibody (Invitrogen, PA1-110), GLUT4 polyclonal rabbit antibody (Invitrogen, PA5-23052), IGF-1 polyclonal goat antibody (Invitrogen, PA5-19382), and Ki67 monoclonal rat antibody (Invitrogen, 14-5698-82) were used. ImageJ was used for immunohistochemical qualitative analysis.

### 2.12. Transmissiovn Electron Microscopy Analyses

The tissue samples were sequentially fixed in 2.5% glutaraldehyde for 2 hours and in 2% osmium acid for 2 hours. The samples were then dehydrated in a gradient of 50%, 70%, 80%, and 90% ethanol for 15 min each (overnight in 70% ethanol) and then three times with 100% ethanol for 20 min each and 2 times with acetone for 15 min each. Maceration, embedding, sectioning (50–70 nm), and staining (3% uranyl acetate–lead citrate double-staining) were performed. Transmission electron microscopy (Jeol, Tokyo, Japan) was used for observation and photography.

### 2.13. Gene Expression Vector Construction

The pcDNA3.1overexpression plasmid was constructed as follows. Briefly, the design of the primers was based on the cDNA sequence of the target gene. Restriction enzymes XhoI and BamHI were inserted at the ends of the open reading frame at the position of the target gene. The specific primers were AR: upstream 5′ TGTAAAACGACGGCCAGT and downstream 5′ CAGGAAACAGCTATGACC; CIP2A: upstream 5′ TGTAAAACGACGGCCAGT and downstream 5′ CAGGAAACAGCTATGACC. The purified PCR products were the target gene linked to the vector pMD18-T using T4 DNA ligase (Takara, Kyoto, Japan) and the constructed pMD18-T-gene plasmid and were sequenced for confirmation. Both the pcDNA3.1 and pMD18-T-gene vectors were digested with BamHI and XhoI, purified and sequenced, and linked to the recombinant pcDNA3.1-gene. PcDNA3.1-gene DNA was confirmed by sequencing.

Lentiviruses carrying shRNAs targeting specific genes (shRNA targeting sequences: CCGGGCCCTACGTCTTCCTTCTATTCTCGAGAATAGAAGGAAGACGTAGGGCTTTTTG) were provided by Sigma-Aldrich. The viruses were expanded in HEK 293T cells and titrated according to the manufacturer’s instructions, and lentiviruses containing non-specific shRNAs (NC-shRNA) were used as controls. Ishikawa cells were infected with purified virus at a 20-fold multiplicity of infection overnight. Each viral suspension was replaced with fresh medium the day after infection, and the expression of the proteins was determined by qRT-PCR and Western blot.

### 2.14. Lentiviral Transfection

Cells were transfected with 100-nM shRNA using Lipofectamine 2000 (Invitrogen) following the instructions. Negative control cells were transfected with Lipofectamine 2000 without shRNA. G418 reagent was used for the selection of transfected cells. qRT-PCR and Western blot were used to determine the mRNA and protein expression in each subset of cells.

### 2.15. Construction of PCOS Mouse Model with Early EC

A PCOS model was established in 4-week-old female mice with the injection of DHEA (ApexBio, Houston, TX, USA, No. B1375, 6 mg/100 g, dissolved in 0.01 mL 95% ethanol) for 28 days. Before that, the mice were transplanted with orthotopic EC. In brief, Ishikawa cells (5 × 10^6^/mL) were injected subcutaneously in the right flanks of nude mice. The tumor mass was removed after 4 weeks and cut into 0.5-mm^3^ sections. The small tumor mass was transplanted into the uterine cavity of mice with surgical procedures; then, the incision was sutured. PCOS was induced after 48-h tumor transplantation. The mice were fed a high-fat diet (60% Kcal fat) during this period. After 28 days, PCOS mouse models with early EC were verified by pathological examination. The therapeutic trial was performed with these models using a combination of Diane-35 (dissolved in 50-mL 1% CMC and administered at 5-mL/kg BW gavage daily) and metformin (500 mg/kg daily by intraperitoneal injection), with a saline solution as the control. After 4 weeks, the animals were sacrificed by cervical dislocation. Mouse endometrial tissue was carefully exfoliated from the uterus under a microscope and stored in liquid nitrogen and formalin fixative. All experiments involving animals were performed following the National Institutes of Health Guide for the Care and Use of Laboratory Animals and were approved by the Institutional Animal Care and Use Committee.

### 2.16. Statistical Analysis

Data were shown as the mean ± standard deviation. The statistical analysis was done by using one-way ANOVA, and *p*-values < 0.05 were considered significant.

## 3. Results

### 3.1. Diane-35 and Metformin Reduced AR and IGF-1 Expression and Increased GLUT4 Expression in Early Stage Tumor Tissue of PCOS Patients with EC

**Figure 1:**
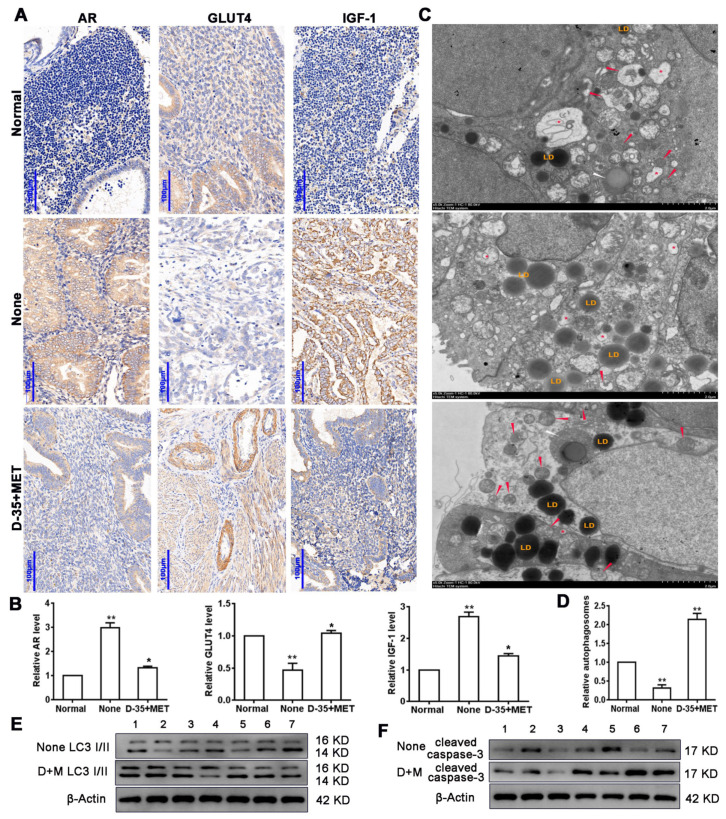
Diane-35 and metformin reduced AR and IGF-1 expression and increased GLUT4 expression in the early-stage tumor tissue of PCOS patients with EC. Immunohistochemistry (**A**) and PCR analysis (**B**) for AR, GLUT4, and IGF-1 in endometrial carcinoma tissue, scale bars = 100 μm. Transmission electron microscope analysis of patients’ tissues: autophagosomes: red arrow, autolysosome: white arrow, lysosome: red asterisk, and lipid droplet: LD, scale bars = 2 μm (**C**,**D**). Western blotting analysis of LC3 I/II (**E**) and cleaved caspase-3 (**F**) expression levels from 7 different patient samples. Data are shown as the mean ± S.D. * *p* < 0.05 and ** *p* < 0.01, ANOVA.

Previous research identified that a combination of Diane-35 and metformin ameliorated the development of early EC in women with PCOS. By using IHC, we assessed the abundance of AR, GLUT4, and IGF-1 in tissue samples from patients who underwent an excision operation. The representative image shows that AR and IGF-1 staining was increased in the EC patients and was attenuated after the combined treatment with Diane-35 and metformin, but GLUT4 had the opposite result (Figure 1A). The PCR analysis revealed a higher level of AR expression but a lower level of GLUT4 expression in patients before therapy. A negative correlation was found to exist between GLUT4 and AR expression. After the combination treatment, the expression of AR was lower, and GLUT4 was increased. The expression of IGF-1 was also lower in tissue samples from those patients who underwent an excision operation after treatment with Diane-35 and metformin when compared to EC patients who were not treated (Figure 1B). Next, we examined autophagy by transmission electron microscope in patient tissues. The results showed that the number of autophagosomes was slightly reduced in early EC epithelial tissues compared to normal tissue but elevated after the Diane-35 and metformain treatment (Figure 1C,D). Western blot analysis of the autophagy marker protein LC3 I/II also revealed increased autophagy level after the combination therapy (Figure 1E). In addition, we detected a high expression of the apoptotic protein, cleaved caspase-3, in EC tissues after the combination therapy (Figure 1F), which indicated that Diane-35 and metformin could repress EC cell proliferation. Taken together, these results demonstrated that Diane-35 and metformin can reduce the symptoms associated with early-stage EC by repressing epithelial cell growth and promoting autophagy.

### 3.2. Diane-35 and Metformin Promote Apoptosis and Autophagy in EC Cells

**Figure 2:**
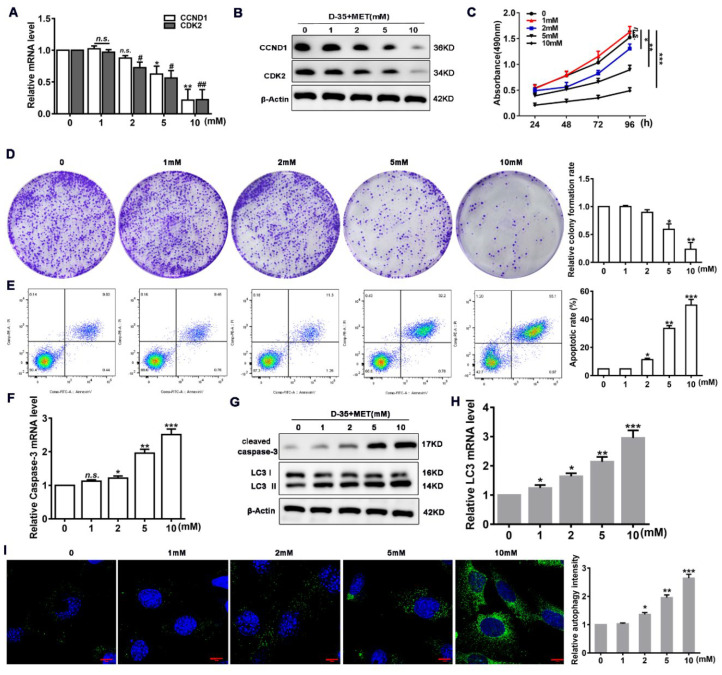
Diane-35 and metformin promote apoptosis and autophagy in EC cells. Human endometrial carcinoma (Ishikawa) cells were treated with increasing concentrations of Diane-35 and metformin (0, 1, 2, 5, and 10 mM) for 24 h. Then, qRT-PCR (**A**) and Western blot (**B**) analyses were used to assess cell proliferation genes CCND1and CDK2 and protein expression. CCK-8 (**C**) and colony formation assay (**D**) were used to evaluate proliferation, and flow cytometry (**E**) was performed to assess apoptosis. Apoptotic biomarker, cleaved caspase-3, was assessed by qRT-PCR (**F**) and Western blot (**G**) analyses. Cell autophagic markers were assessed by qRT-PCR (**H**), Western blotting (**G**), and immunofluorescence staining for LC3 (green) and DAPI (blue) (**I**). Data are shown as the mean ± S.D., *n* = 3. *n.s.* = no statistically significant differences. * *p* < 0.05, ^#^ *p* < 0.05, ** *p* < 0.01, ^##^ *p* < 0.01 and *** *p* < 0.001, ANOVA. Scale bars = 10 μm.

To verify whether Diane-35 and metformin have an impact on EC, we treated human EC (Ishikawa) cells with increasing concentrations of Diane-35 and metformin for 24 h and then measured the levels of proliferation. To measure proliferation and apoptosis, we used the cell division markers cyclin D and CDK2 and the apoptosis marker cleaved, caspase-3. The RNA expression and protein levels of cyclin D and CDK2 decreased concentration-dependently (Figure 2A,B). At Diane-35 and metformin concentrations of 10 mM, cyclin D and CDK2 RNA expression decreased by more than 60%. Correspondingly, the cell viability and colony formation also decreased (Figure 2C,D), whereas the levels of apoptosis, measured by flow cytometry and the apoptotic marker cleaved, caspase-3, increased (Figure 2E–G). The levels of cell autophagy detected by the LC3-I/II protein rose with increased concentrations of Diane-35 and metformin (Figure 2G,H). Autophagy was shown to increase considerably in LC3-targeted immunofluorescence images of Ishikawa cells treated with high concentrations of Diane-35 and metformin (Figure 2I). These results are consistent with the tissue detection results and confirmed that the combination of Diane-35 and metformin can inhibit proliferation and promote apoptosis and autophagy in EC cells.

### 3.3. Diane-35 and Metformin Repress Apoptotic and Autophagy in Ovarian Granulosa Cells of a PCOS Murine Model

**Figure 3:**
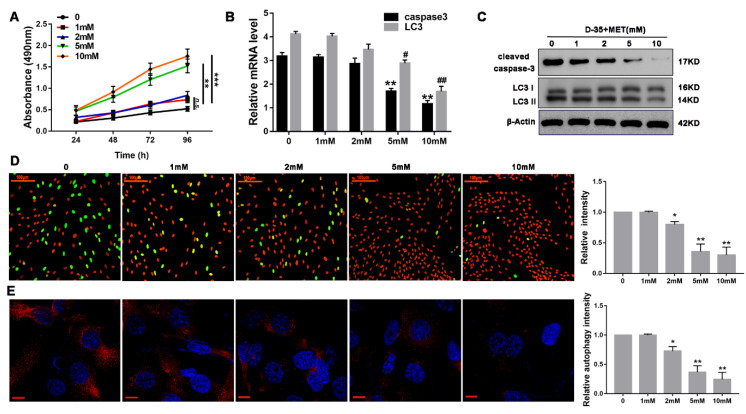
Diane-35 and metformin repressed ovarian granulosa cells apoptosis in a murine model of PCOS. Ovarian granulosa cells were isolated from mice ovaries. Then, cells were treated with increasing concentrations of Diane-35 and metformin (0, 1, 2, 5, and 10 mM) for 24 h. A CCK-8 assay was performed to measure cell viability (**A**). RT-PCR (**B**) and Western blotting (**C**) were used to detect cleaved caspase-3 and LC3 expression in granulosa cells. Granulosa cells apoptosis was determined by the Tunel assay, scale bars = 100 μm (**D**). Immunofluorescence assay was performed using LC3 I/II antibody, red for LC3, scale bars = 10 μm (**E**). Data are shown as the mean ± S.D., *n* = 3. *n.s.* = no statistically significant differences. * *p* < 0.05, ^#^ *p* < 0.05, ** *p* < 0.01, ^##^ *p* < 0.01 and *** *p* < 0.001, ANOVA.

To demonstrate that the combination Diane-35 and metformin treatment improves ovarian development in PCOS, we constructed a PCOS mouse model, isolated ovarian granulosa cells, and cultured them in vitro. Ovarian granulosa cells are reported to have increased autophagy and apoptosis in PCOS patients [29]. We treated PCOS ovarian granulosa cells with different concentrations of Diane-35 and metformin. Interestingly, the CCK-8 assay showed that cell viability increased with the increasing concentrations (Figure 3A). PCR and Western blot analyses for cleaved caspase-3 and Tunel fluorescence showed that cell apoptosis decreased with the increasing concentrations of Diane-35 and metformin (Figure 3B–D). In the cellular autophagy study, the results of the PCR and Western blot detection of autophagy marker molecule LC3 I/II and immunofluorescence assay showed that the autophagic activity in PCOS ovarian granulosa cells decreased after treatment (Figure 3B,C,E), indicating that the combination treatment inhibited the abnormal activation of autophagic activity in ovarian granulosa cells of PCOS. The above results suggest that Diane-35 and metformin not only inhibit the proliferation of endometrial cancer epithelial cells and increase cellular autophagy but also significantly improve ovarian granulosa cell development in PCOS by inhibiting apoptosis and abnormal autophagic activity.

### 3.4. Cell Proliferation and Insulin Resistance Is Regulated by Diane-35 and Metformin

**Figure 4:**
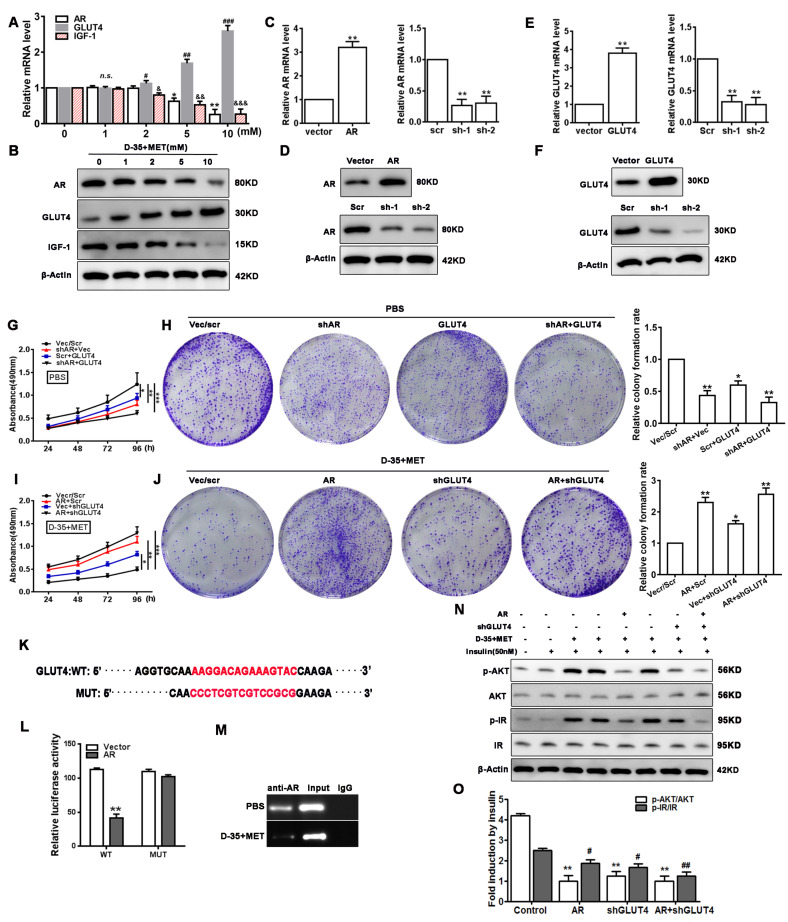
Cell proliferation and insulin resistance is regulated by Diane-35 and metformin. Ishikawa cells were treated with increasing concentrations of Diane-35 and metformin for 24 h (0, 1, 2, 5, and 10 mM). Then, the expression of AR, GLUT4, and IGF-1 was assessed by qRT-PCR (**A**) and Western blotting (**B**). qRT-PCR (**C**,**E**) and Western blot (**D**,**F**) analyses were done for transfection efficiency measurements of the GLUT4 and AR expression vectors in Ishikawa cells. CCK-8 and colony formation assays were performed in transfected cells treated with (**G**,**H**) or without (**I**,**J**) Diane-35 and metformin (10 mM). (**K**) Wild and mutant promoter sequences of the GLUT4 gene. (**L**) Dual-luciferase reporting experiment for AR. (**M**) ChIP assay for binding validation between the AR and GLUT4 promoters. (**N**,**O**) Under Diane-35 and metformin treatment, p-AKT, AKT, p-IR, and IR expression levels in AR, and GLUT4-transfected Ishikawa cells with insulin stimulation. Data are shown as the mean ± S.D., *n* = 3. *n.s.* = no statistically significant differences. * *p* < 0.05, ^#^ *p* < 0.05, ^&^ *p* < 0.05, ** *p* < 0.01, ^##^ *p* < 0.01, ^&&^ *p* < 0.01 and *** *p* < 0.001, ^###^ *p* < 0.001, ^&&&^ *p* < 0.001, ANOVA.

To confirm the effect of Diane-35 and metformin on the expression of AR, GLUT4, and IGF-1, we treated Ishikawa cells with increased concentrations of Diane-35 and metformin and then measured the expression and protein levels by qRT-PCR and Western blotting, respectively (Figure 4A,B). The levels of AR and IGF-1 expression decreased with increasing concentrations of Diane-35 and metformin, whereas the levels of GLUT4 increased, indicating that there was a concentration-dependent reduction in insulin resistance in EC cells, which coincided with the clinical histological examination. To explore the role of AR and GLUT4 in cell growth, we constructed AR and GLUT4 stable overexpression and knockdown cell lines using lentiviral expression vectors in Ishikawa cells (Figure 4C–F). Then, we conducted CCK -8 and colony formation assays using the EC cell lines. The results showed that the cell viability and colony number were decreased with AR knockdown or GLUT4 overexpression without Diane-35 and metformin treatment (Figure 4G,H). However, the cell viability and colony number were significantly increased with AR overexpression and GLUT4 knockdown, even with the addition of Diane-35 and metformin (Figure 4I,J). In a further study of the relationship between AR and GLUT4, a dual-luciferase reporting experiment was performed with wild and mutant GLUT4 promoter sequences. The results indicated that AR interacted with the promoter of GLUT4, which was confirmed by ChIP assays with the AR antibody (Figure 4K–M). These data indicated that AR could inhibit GLUT4 expression via the specific binding of the promoter, and the Diane-35 and metformin combination inhibited AR expression, thereby promoting an increase in GLUT4 and a decrease in EC cell proliferation.

To elucidate the regulation of the insulin signaling pathway by AR via GLUT4, we assessed the insulin resistance through the measurement of the AKT pathway and IR (insulin receptor) activation in EC cells transfected with AR or (and) GLUT4 expression vectors under Diane-35 and metformin treatment and insulin stimulation (50 nM). The results showed that the treatment of Diane-35 and metformin significantly promoted the AKT pathway and IR activation but were repressed by AR overexpression and GLUT4 knockdown (Figure 4N,O). These results implied that Diane-35 and metformin regulate EC cell insulin resistance via AR and GLUT4.

### 3.5. Autophagy Is Induced by Diane-35 and Metformin via Inhibition of IGF-1

**Figure 5:**
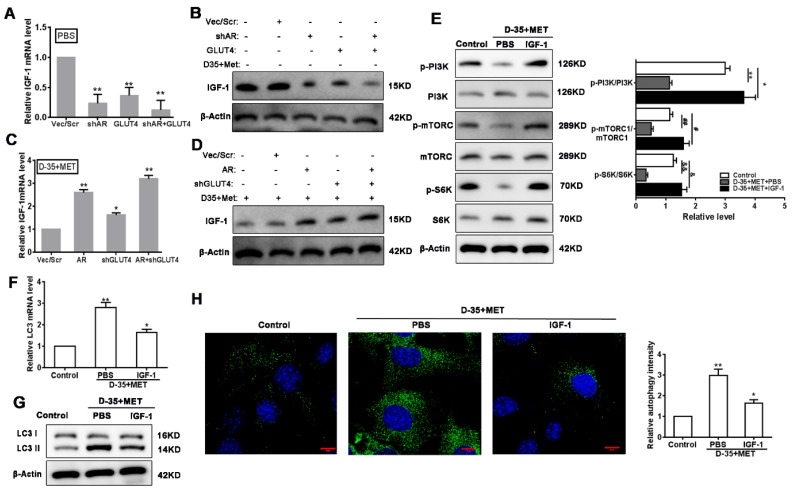
Autophagy is enhanced by inhibition of the IGF-1-mediated PI3K/mTORC1 pathway. IGF-1 expression level in Ishikawa cells with the transfection of different AR and GLUT4 expression vectors with or without Diane-35 and metformin treatment (**A**–**D**). Effects of Diane-35 and metformin (10 mM) on the expression of the PI3K, AKT, and mTORC1 pathways in Ishikawa cells with or without the addition of IGF-1 (**E**). Cell autophagy was measured using qRT-PCR (**F**), Western blotting (**G**), and immunofluorescence (**H**). Data are shown as the mean ± S.D. * *p* < 0.05, ^#^ *p* < 0.05, ^&^ *p* < 0.05 and ** *p* < 0.01, ^##^ *p* < 0.01, ^&&^ *p* < 0.01, ANOVA. Scale bars = 10 μm.

To elucidate the specific mechanism of autophagy in EC cells induced by Diane-35 and metformin treatment, we first investigated the effect of AR and GLUT4 on IGF-1 expression. We found that both AR knockdown and GLUT4 overexpression significantly reduced the IGF-1 levels in the absence of Diane-35 and metformin intervention (Figure 5A,B); however, we determined that Diane-35 and metformin treatment reduced IGF-1 expression in EC cells (Figure 4A,B), but the IGF-1 expression level was obviously increased by either AR overexpression or (and) GLUT4 knockdown (Figure 5C,D). It has been reported that IGF-1 regulates cell autophagy via the PI3K/mTORC pathway [21]. Hence, we assessed whether the reduction in IGF-1 expression through Diane-35 and metformin therapy could influence the PI3K/mTORC1 pathway. The levels of PI3K, mTORC1, and S6K phosphorylation were measured in Ishikawa cells treated with Diane-35 and metformin with or without the addition of IGF-1. The data showed that Diane-35 and metformin therapy inhibited the activation of the PI3K/mTORC1 pathway; however, the addition of IGF-1 activated the PI3K/mTORC1 pathway and S6K level (Figure 5E). Then, we measured the level of autophagy in the same cells by determining the conversion of microtubule-associated protein 1 light chain 3 (LC3)-I to the phosphatidylethanolamine-conjugated form LC3-II, which is integrated into the membranes of autophagosomes [30,31]. Diane-35 and metformin increased autophagy, whereas IGF-1 inhibited autophagy (Figure 5F–H). These results indicated that Diane-35 and metformin increased cellular autophagy by inhibiting the activation of IGF-1 on the PI3K/mTORC1 pathway.

### 3.6. Verification of PI3K/mTORC1 Pathways on Diane-35 and Metformin-Mediated Autophagy

**Figure 6:**
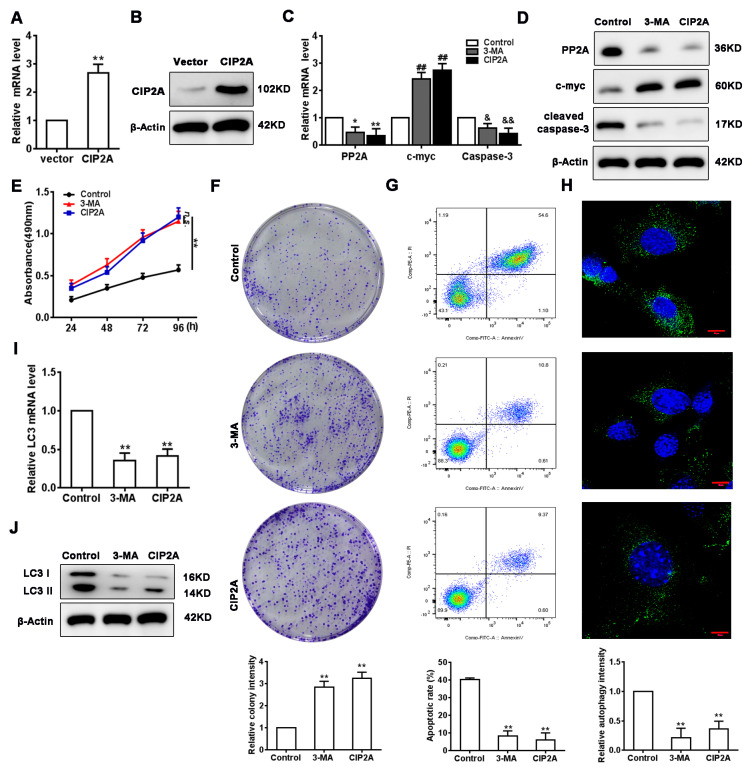
Verification of the PI3K/mTORC1 pathway on Diane-35 and metformin-mediated autophagy. (**A**,**B**) Transfection efficiency of the CIP2A overexpression system in Ishikawa cells. (**C**,**D**) mRNA and protein levels of PP2A, c-Myc, and cleaved caspase-3 in cells subjected to different treatments. CCK-8 (**E**), colony formation (**F**), and flow cytometry (**G**) were performed to measure apoptosis in Ishikawa cells after pretreatment with 3-methyladenine (3-MA, 5mM) or transfection with the CIP2A overexpression vector. All the cells were treated with Diane-35 and metformin (10 mM). (**H**–**J**) Cell autophagy was determined using the LC3 level measured by qRT-PCR, Western blotting, and immunofluorescence. Data are shown as the mean ± S.D. *n.s.* = no statistically significant differences. * *p* < 0.05, ^&^ *p* < 0.05, and ** *p* < 0.01, ^##^ *p* < 0.05, ^&&^ *p* < 0.01, ANOVA. Scale bars = 10 μm.

Next, we verified that Diane-35 and metformin induce autophagy in EC cells via the PI3K/mTORC1 pathway. As reported, the inhibition of autophagy by mTORC1 in EC is thought to be activated by CIP2A; therefore, we measured its involvement in the regulation of autophagy by measuring the expression of the autophagic marker PP2A [31] and cell proliferation and apoptotic indicators (c-Myc and cleaved caspase-3) in Ishikawa cells treated with Diane-35 and metformin. Meanwhile, the PI3K pathway was blocked by the inhibitor, 3-methyladenine (3-MA). First, we constructed CIP2A overexpression lentiviral vectors and then transfected Ishikawa cells (Figure 6A,B). The overexpression of CIP2A in Ishikawa cells led to the downregulation of PP2A and cleaved caspase-3 (increased with Diane-35 and metformin treatment) and the upregulation of c-Myc (Figure 6C,D). Moreover, 3-MA had the same effect on the expression of PP2A, c-Myc, and cleaved caspase-3 (Figure 6C,D). Furthermore, there was an increase in cell proliferation and viability when CIP2A was overexpressed or when using 3-MA (Figure 6E,F), which also declined during apoptotis by flow cytometry (Figure 6G) and coincided with cleaved caspase-3 and c-myc expression. Cell autophagy was repressed by the PI3K pathway inhibitor 3-MA and mTORC1 upstream inhibitor molecule CIP2A, as shown by qRT-PCR, Western blotting, and immunofluorescence (Figure 6H–J). Overall, the results demonstrated that the PI3K/mTORC1 pathway is responsible for the IGF-1-induced activation of autophagy in EC cells mediated by Diane-35 and metformin.

### 3.7. Diane-35 and Metformin Administration Ameliorate EC In Vivo

**Figure 7:**
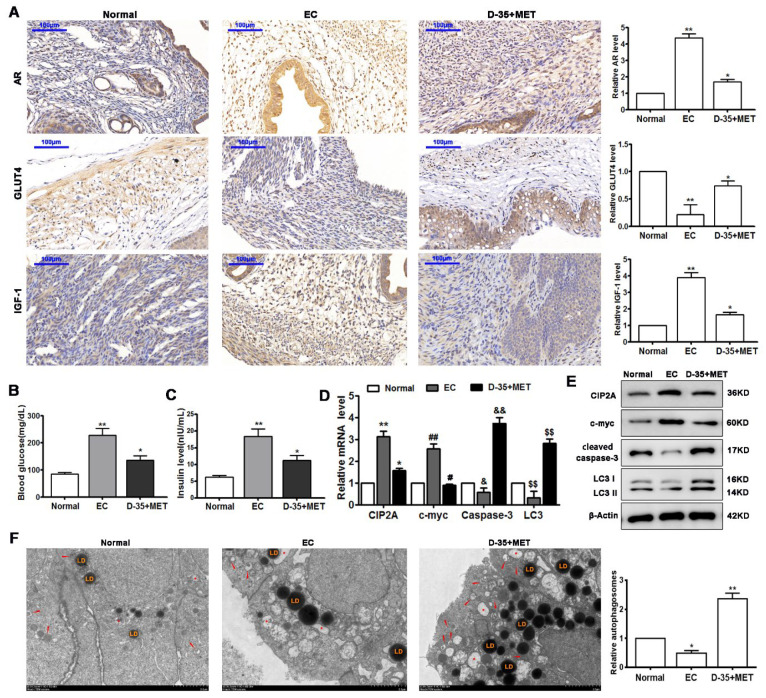
Diane-35 and metformin administration ameliorated EC in vivo. An orthotopic xenograft model was established in nude mice. Endometrium tissues of the model in mice were obtained for the immunohistochemistry analysis (**A**), scale bars = 100 μm. Insulin resistance was assessed by using the blood glucose level (**B**)and blood insulin level (**C**). CIP2A, c-myc, cleaved caspase-3, and LC3 I/II mRNA (**D**) and protein (**E**) expression. Transmission electron microscope analysis of patients’ tissues. Autophagosomes: red arrow, autolysosome: white arrow, lysosome: red asterisk, and lipid droplet: LD, scale bars = 2 μm (**F**). Data are shown as the mean ± S.D. * *p* < 0.05, ^#^ *p* < 0.05, ^&^ *p* < 0.05 and ** *p* < 0.01, ^##^ *p* < 0.01, ^&&^ *p* < 0.01, ^$$^ *p* < 0.01, ANOVA.

To discover if Diane-35 and metformin had a similar influence in vivo, we repeated the experiments in an animal model of PCOS using nude mice with early-stage EC established in situ. In endometrium tissue sections, the expression of AR and IGF-1 expression were significantly upregulated in the EC model, whereas GLUT4 was significantly downregulated (Figure 7A). In response to Diane-35 and metformin, the results were inversed, which coincided with the results obinted from the tissue of the patients. Insulin resistance measured by the blood glucose and insulin levels was higher in untreated PCOS-EC mice than in those treated with Diane-35 and metformin (Figure 7B,C). The levels of autophagy and cell apoptosis were determined by measuring the expression of CIP2A, c-Myc, cleaved caspase-3, and LC3 (Figure 7D,E). The expression of CIP2A and c-Myc was increased in the EC model, whereas the autophagic proteins cleaved caspase-3 and LC3 were reduced. However, the administration of Diane-35 and metformin significantly increased the levels of cleaved caspase-3 and LC3, whereas the levels of CIP2A and c-Myc were reduced, suggesting that autophagy and apoptosis were significantly enhanced in response to Diane-35 and metformin. Furthermore, transmission electron microscopy was used to observe the autophagy level in the tissue; the images indicated that the autophagosomes increased after the Diane-35 and metformin treatment (Figure 7F). These results demonstrated that the combination of Diane-35 and metformin improved early-stage EC in an orthotopic xenograft mouse model.

## 4. Discussion

PCOS patients are believed to have an increased risk in EC, but the association between the two conditions remains unclear [32,33]. In this study, we investigated the molecular mechanism behind the combined treatment of Diane-35 and metformin in alleviating the symptoms of early EC. We found that the combined treatment could inhibit the proliferative activity of EC cells and promote apoptosis and autophagy. The level of GLUT4 increased after treatment, which resulted in increased insulin sensitivity and a decreased level of IGF-1. We found that upregulating AR could increase the level of IGF-1 and further established that AR interacted with GLUT4 in the luciferase-reporter and ChIP studies, which was supported by findings in other studies [21,34]. Li et al. [21] found that changes in the expression of GLUT4 in the endometrial tissue of PCOS patients involved AR expression and the IR/PI3K/AKT/mTOR pathway. Correspondingly, we found that the activation of AKT and IR increased the most with insulin stimulation or the treatment with Diane-35 and metformin combined, but the phosphorylation of AKT and IR was decreased when AR was overexpressed or GLUT4 was underexpressed, suggesting that the negative regulation of IR/AKT associated with PCOS was influenced by hyperandrogenism, which can be controlled by Diane-35 and metformin. The results we obtained in the Ishikawa cells in vitro were also demonstrated in patients with PCOS. The levels of AR and IGF-1 expression were reduced, and the level of GLUT4 increased in patients after receiving Diane-35 and metformin for 6 months. Metformin, in particular, is thought to upregulate GLUT4 and is used to treat insulin resistance in PCOS patients [20].

There are many conflicting results about the relationship between autophagy and cancer [24,35,36,37]. Autophagy is thought to promote adaptation in EC cells by increasing the resistance to targeted therapy. For instance, sorafenib is effective in several cancers, but only modest results are shown in EC, which is believed to be a consequence of autophagic resistance. However, isoliquiritigenin was found to promote apoptosis and autophagy in mice by enhancing the protein expression of caspase-7/LC3 and inhibiting cell growth in EC [37]. In addition, metformin is reported to have opposing roles in autophagy. In one study, metformin was found to increase the cell sensitivity to therapy in EC through targeting autophagy [36]. However, metformin has also been found to promote autophagy and apoptosis in esophageal squamous cell carcinoma by the downregulation of Stat3 signaling [24]. Metformin is also believed to have an inhibitory effect on EC through targeting the adenosine monophosphate-activated protein kinase (AMPK)/mTOR and mitogen-activated protein kinase (MAPK) pathways [38]. Similarly, macranthoside B, a saponin compound from *Lonicera macranthoides*, could induce cell death and enhances autophagy in ovarian cancer through the AMPK/mTOR pathway by a mechanism thought to involve the accumulation of reactive oxygen species [39].

In this study, we found that Diane-35 and metformin mediated cellular autophagy and cell death by regulating the PI3K/mTORC1 pathway through the inhibition of IGF-1. The inhibition of autophagy by mTORC1 in EC is thought to involve CIP2A [26]. The overexpression of CIP2A or 3-MA treatment, an inhibitor of autophagy, downregulated PP2A and cleaved caspase-3 and upregulated c-Myc. Consequently, a reduction in autophagy led to an increase in the proliferation and viability of EC cells. Moreover, the results we obtained in vitro were replicated in a mouse model of EC with PCOS. The cleaved caspase-3 and LC3 levels were significantly higher in mice administered Diane-35 and metformin, whereas levels of CIP2A and c-Myc were reduced, suggesting that autophagy was enhanced. The expression of IGF-1 was lower in the Diane-35 and metformin-treated model, indicating that cellular autophagy is regulated by the PI3K/mTORC pathway. CIP2A promotes cancer by suppressing the activity of PP2A [40]. Low levels of growth stimulators, such as IGF-1, reduce the activity of mTORC and induce the autophagic degradation of CIP2A; thereby, autophagy and cell death are increased [41]. Therefore, Diane-35 and metformin may function by reducing the levels of IGF-1.

In agreement with our results, a recent study found that, when autophagy is inhibited, the ability of metformin to induce cell death is decreased, implicating that the antitumorigenic function of metformin is through autophagic cytotoxicity [42]. However, whether the combination of Diane-35 and metformin has a greater effect than them individually may need further study. It is possible that the regulation of autophagy may be primarily through metformin, whereas Diane-35 may primarily regulate the levels of androgens. Several studies have found that Diane-35 combined with metformin attenuates the symptoms of PCOS more than Diane-35 used alone [28,43,44,45]. There were some limitations to our study. We followed through with the results from a preliminary study; therefore, the sample population was taken from patients with PCOS and EC from one institute. In addition, for ethical reasons, the control measurements in the patients could not be maintained for the full duration of the experiment. A future study with a larger and more diverse population would be able to substantiate the results.

## 5. Conclusions

In summary, we found that the combination of Diane-35 and metformin to treat patients with PCOS and EC involved mechanisms that inhibited cell proliferation and ameliorated insulin resistance by repressing the expression of AR, which mediated the onset of cellular autophagy and apoptosis by regulating the PI3K/mTORC pathway through the inhibition of IGF-1. Our results support the role of Diane-35 and metformin as a combined therapy to treat women with PCOS and EC.

## Figures and Tables

**Table 1:** Primers used in the study.

AR	Forward: 5′ AGGATGCTCTACTTCGCCCC Reverse: 5′ CTGGCTGTACATCCGGGAC
GLUT4	Forward: 5′ CAGCTCTCAGGCATCAAT Reverse: 5′ TCTACTAAGAGCACCGAG
IGF-1	Forward: 5′ CCATAGAAAGAGGAATAACAGCAG Reverse: 5′ TACCTCCCATTCATCAGGCA
CIP2A	Forward: 5′ GAACAGATAAGAAAAGAGTTGAGCATT Reverse: 5′ CGACCTTCTAATTGTGCCTTTT
PP2A	Forward: 5′ CACACGGACCAAAAGATGTGCC Reverse: 5′ CAGCACCAGTCGTGCCCACTG
LC3	Forward: 5′ AGCAGCATCCAACCAAAATC Reverse: 5′ CTGTGTCCGTTCACCAACAG
Caspase-3	Forward: 5′ CAGTGGAGGCCGACTTCTTG Reverse: 5′ TGGCACAAAGCGACTGGAT
CDK2	Forward: 5′ CCAGGAGTTACTTCTATGCCTGA Reverse: 5′ TTCATCCAGGGGAGGTACAAC
CCND1	Forward: 5′ GCTGCGAAGTGGAAACCATC Reverse: 5′ CCTCCTTCTGCACACATTTGAA
GAPDN	Forward: 5′ CGGGGCTGGCATTGCTCTC Reverse: 5′ GGGGCCGACTTGGGATAGG

## Data Availability

The data presented in this study are available on request from the corresponding author.

## Abbreviations

PCOS	polycystic ovary syndrome
EC	endometrial carcinoma
AP	androgen receptor
AMP	adenosine monophosphate
AMPK	adenosine monophosphate-activated protein kinase
GLUT4	Glucose transporter 4
IGF-1	insulin-like growth factor
mTORC	rapamycin complex
3-MA	3-methyladenine
